# Associations, overlaps and dissociations between apathy and fatigue

**DOI:** 10.1038/s41598-022-11071-5

**Published:** 2022-05-05

**Authors:** Lyne Daumas, Camille Corbel, Raphaël Zory, Xavier Corveleyn, Roxane Fabre, Philippe Robert, Valeria Manera

**Affiliations:** 1 Université Côte d’Azur, CoBTeK Lab, Nice, France; 2 Université Côte d’Azur, LAMHESS Lab, Nice, France; 3 Université Côte d’Azur, LAPCOS Lab, Nice, France; 4 Institut Universitaire de France, Paris, France; 5 Public Health Department, Université Côte d’Azur, Nice University Hospital, Nice, France

**Keywords:** Health care, Signs and symptoms

## Abstract

Apathy and fatigue have a high prevalence in many pathological populations, but they are also present in healthy adults. The relationship between apathy and fatigue, which are both multidimensional, is still poorly understood. This study aims to describe the associations between the subdimensions of both apathy and fatigue and to investigate their overlaps and dissociations in healthy people. 729 participants (mean age = 30.8 ± 10.7 years) completed online self-assessment questionnaires. The Apathy Motivation Index and Dimensional Apathy Scale were used to assess apathy. The Multidimensional Fatigue Inventory was used to assess fatigue. The executive dimension of apathy showed the strongest correlations with mental fatigue and the two appeared to be underpinned by the same latent factor, according to exploratory factor analysis (EFA). The factor structure of EFA showed overlaps between behavioral apathy and both reduced motivation and activity in fatigue. Emotional and social dimensions of apathy were separately underpinned by a latent factor that comprised no items of fatigue. Apathy and fatigue have reduced activity and mental difficulties in common, whereas emotional and social disorders distinguish apathy from fatigue. This has important implications for assessing apathy and fatigue in the general population, and may be relevant for clinical practice.

## Introduction

Apathy is a clinical syndrome characterized by a reduction in self-initiated, goal-directed activity that is not driven by a primary motor or sensory impairment^1^. It is not a unitary syndrome but can manifest across several domains^2^, notably cognition, behavior, emotion and social interaction^3^. Although it occurs frequently in several neurological and psychiatric disorders, it is also apparent to varying degrees in healthy people^3,4^. Apathy in healthy adults significantly affects everyday life, particularly with regard to education and employment opportunities^5,6^. Some forms of apathy can be linked to psychopathy traits and a lack of empathy^4^, which suggests the importance of also assessing apathy in the general population. The overall reduction of goal-directed behavior is not specific to apathy but can also result from other conditions, such as fatigue. Fatigue may refer on the one hand, to the subjective sensations of lack of physical or mental energy, and on the other hand, to the reduced ability to maintain optimally a motor or cognitive work for a long time^7,8^. Similar to apathy, fatigue can be described as a multidimensional disorder, which includes physical and mental dimensions that manifest by unpleasant sensations and/or limitations in execution of behavioral or cognitive activities. Assessment and treatment of fatigue are complex because of its multifactorial nature, and because fatigue can be observed with or without underlying medical illness. Fatigue is commonly experienced in the general population, including healthy adults. It mostly manifests as a temporary state but can also be persistent. In approximately 20% of healthy individuals, fatigue is chronic and has a major impact on everyday functioning^9,10^. So, both apathy and fatigue manifest in difficulty in starting or maintaining the usual or desired activities, and evidence suggests that for both there is a perturbation in effort-based decision-making, which results in lower willingness to engage in effortful behavior^11–13^.

Scientific definitions of apathy include a “lack of energy” and, conversely, fatigue is sometimes described as a “lack of motivation,” indicating a conceptual overlap between the two. In addition, apathy and fatigue may often be confused due to their similar manifestations and repercussions. They are nevertheless two distinct conditions that may occur alone or be concomitant^14,15^. Despite their negative impact on healthy people and their clinical relevance in pathological populations, the exact relationships between apathy and fatigue are still poorly understood, and differentiating them is sometimes difficult. Apathy and fatigue are both multidimensional concepts, and their various subdimensions might be differently related to each other and overlap to some extent. So far, two studies have already conducted correlational analyses between apathy and fatigue subscales^3,11^. Interestingly, they found significant links between all apathy and fatigue subscores, with positive correlations between fatigue and behavioral apathy, and mainly negative correlations between fatigue and emotional apathy.

The aim of the present study was twofold: to describe the associations between the subdimensions of apathy and fatigue through correlation analyses, and to investigate their overlaps and dissociations through exploratory factor analyses (EFA) in a representative sample of healthy participants. The EFA allows to explore the structure of the relationships between variables and allows to identify latent factors. The Apathy Motivation Index (AMI) and Dimensional Apathy Scale (DAS) were used to assess apathy^3,16^. The Multidimensional Fatigue Inventory (MFI) was used to assess fatigue^17^. We hypothesized that the co-occurrence of apathy and fatigue would be underpinned by common latent factors, although they would also be distinguishable on other dimensions, confirming the importance of differentiating them in the general population. Results may have also important clinical implication for pathological population such as in neurocognitive disorders and Parkinson disease, where apathy and fatigue are highly prevalent.

## Results

In all, 729 participants aged from 18 to 68 years (mean age = 30.8 years ± 10.7) were included in the study. Total AMI scores ranged from 0.39 to 3.11 with a mean of 1.53 ± 0.45. Total DAS scores ranged from 9 to 50 with a mean of 38.0 ± 4.59. Total MFI scores ranged from 20 to 100 with a mean of 56.55 ± 18.15. The range, the average, the standard deviation of subscales, the skewness of data (see Supplementary Table S1) and frequency histograms are provided in supplementary materials (see Supplementary Fig. S1).

### Relationship between apathy and fatigue domains

**Figure 1:**
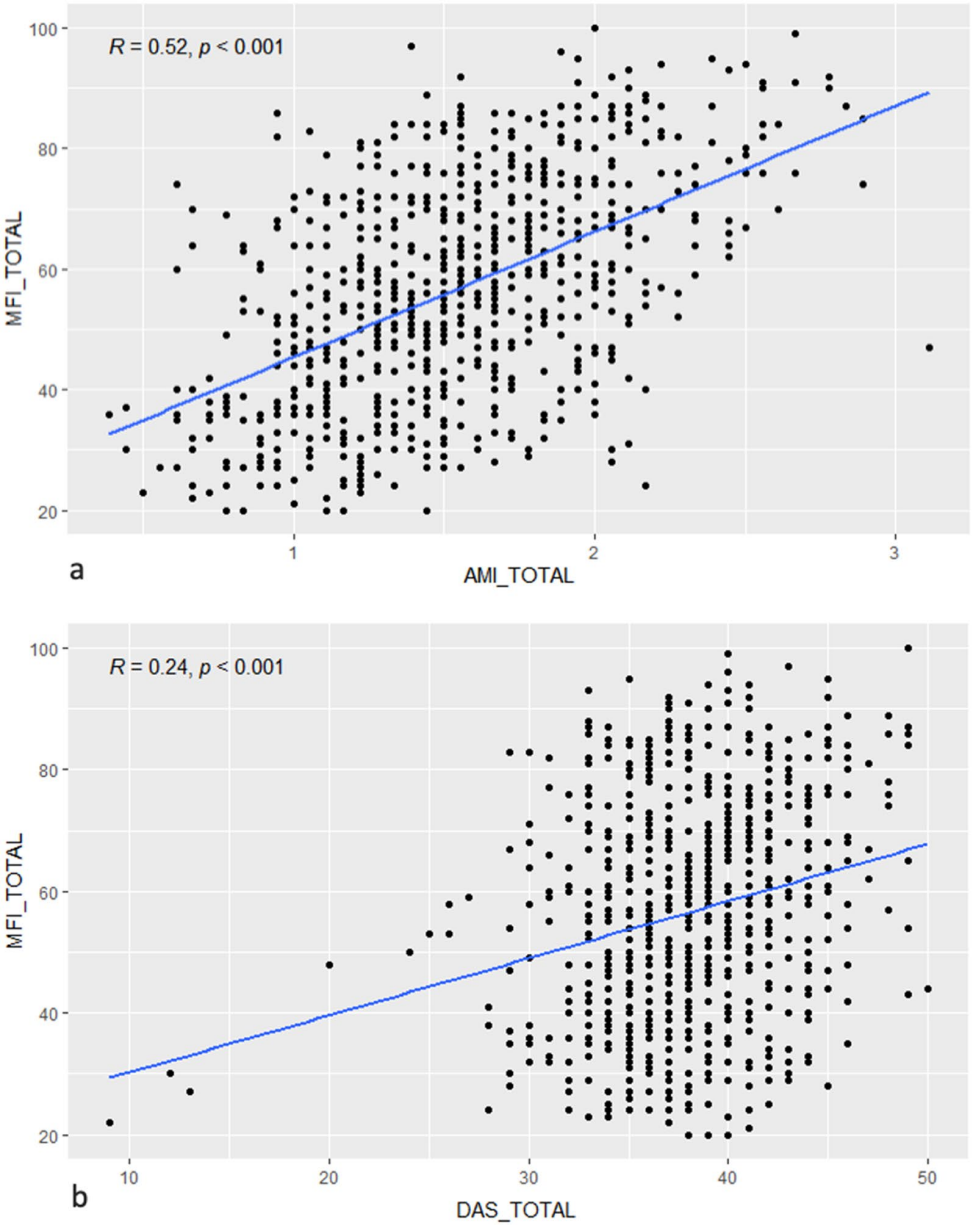
Association between fatigue and apathy: correlations between total MFI score and (**a**) total AMI score and (**b**) total DAS score.

Correlational analyses between the overall and subscale scores of the apathy and fatigue questionnaires using Spearman’s test are shown in Table 1. Results from Pearson’s tests, showing the same pattern of results, are provided in the supplementary materials (see Supplementary Table S2). Total MFI scores showed positive correlations with both AMI (*r* = 0.52, *p* < 0.001, Fig. 1a) and DAS total scores (*r* = 0.24, *p* < 0.001, Fig. 1b). For the AMI, the behavioral and social subscores were positively correlated with all subscores of MFI, whereas the emotional subscale was negatively correlated with the MFI subscales. The highest correlations were found between the behavioral component and the total MFI score (*r* = 0.63, *p* < 0.001), reduced activity (*r* = 0.62, *p* < 0.001), reduced motivation (*r* = 0.55, *p* < 0.001), and mental fatigue (*r* = 0.54, *p* < 0.001). For the DAS, the initiation and executive subscores showed positive correlations with all subscores of MFI. The highest correlations were found between the executive subscore and mental fatigue (*r* = 0.77, *p* < 0.001), the total MFI score (*r* = 0.70, *p* < 0.001), reduced motivation (*r* = 0.64, *p* < 0.001), and reduced activity (*r* = 0.60, *p* < 0.001).

**Table 1:** Spearman’s correlations of MFI subscales with AMI and DAS subscales.

Apathy questionnaires	AMI				DAS			
	ES	BA	SM	Total	EMO	INIT	EXE	Total
**Multidimensional fatigue inventory (MFI)**								
GF	− 0.259* (0.000)	0.376* (0.000)	0.324* (0.000)	0.292* (0.000)	− 0.079 (0.031)	0.248* (0.000)	0.425* (0.000)	0.080 (0.030)
PF	− 0.193* (0.000)	0.436** (0.000)	0.371* (0.000)	0.392* (0.000)	0.015 (0.678)	0.328* (0.000)	0.438* (0.000)	0.116 (0.002)
MF	− 0.277* (0.000)	0.531* (0.000)	0.309* (0.000)	0.371* (0.000)	0.017 (0.654)	0.283* (0.000)	0.777* (0.000)	0.195* (0.000)
RA	− 0.197* (0.000)	0.609* (0.000)	0.407* (0.000)	0.515* (0.000)	0.103 (0.005)	0.476* (0.000)	0.601* (0.000)	0.266* (0.000)
RM	− 0.241* (0.000)	0.537* (0.000)	0.416* (0.000)	0.448* (0.000)	0.026 (0.487)	0.436* (0.000)	0.633* (0.000)	0.195* (0.000)
Total	− 0.272* (0.000)	0.613* (0.000)	0.452* (0.000)	0.505* (0.000)	0.039 (0.292)	0.446* (0.000)	0.690* (0.000)	0.208* (0.000)

*AMI* apathy motivation index, *BA* behavioral activation, *ES* emotional sensitivity, *SM* social motivation, *DAS* dimensional apathy scale, *EXE* executive, *INIT* initiation, *EMO* emotional, *MFI* multidimensional fatigue inventory, *GF* general fatigue, *PF* physical fatigue, *MF* mental fatigue, *RA* reduced activity, *RM* reduced motivation.

*Correlation with *p* < 0.001.

### Overlap and distinction between apathy and fatigue

**Figure 2:**
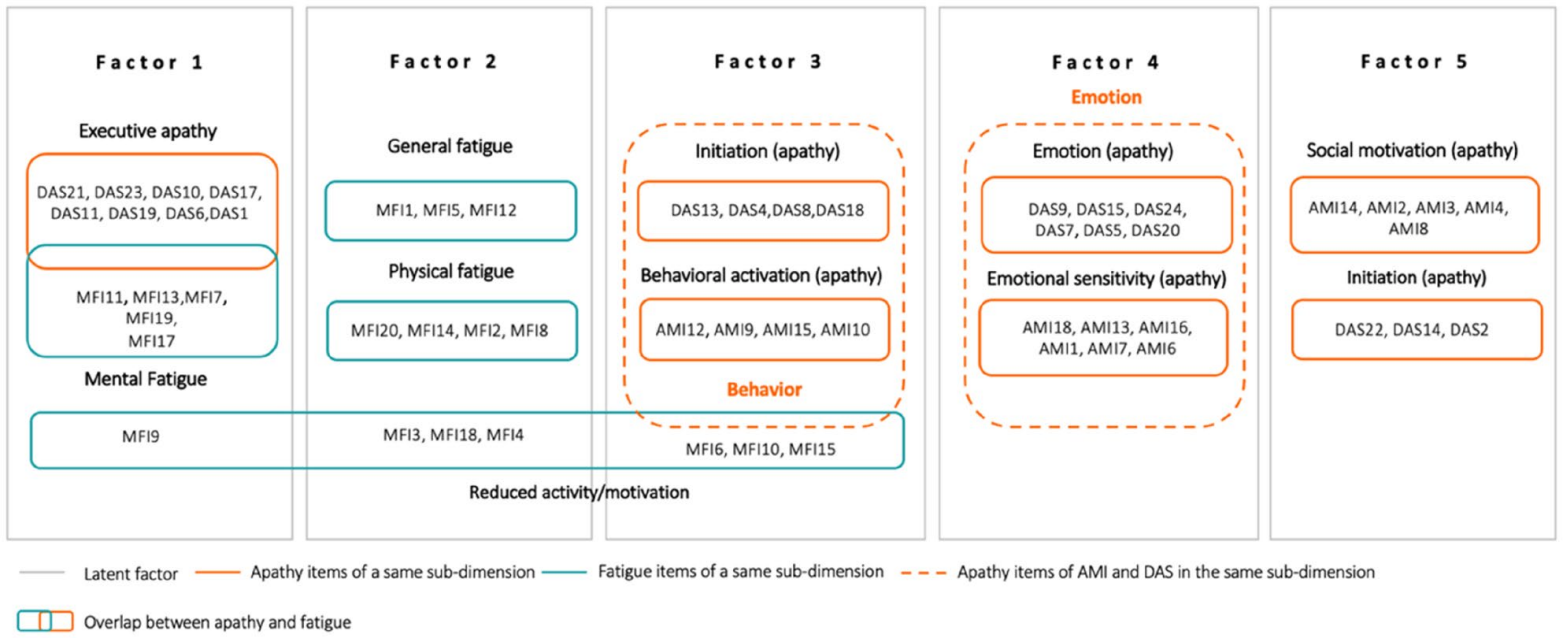
Schematic five-factor structure. The five gray boxes correspond to the five latent factors. Items inside a gray box means that they are supported by the corresponding factor. Blue boxes contain MFI items. Orange boxes with full lines contain items from apathy questionnaires belonging to the same subdimension (e.g., executive apathy, behavioral activation). Orange boxes with dashed lines include DAS and AMI items belonging to the same apathy subdomain. We can see overlapping between apathy and fatigue when the blue and orange boxes touch each other.

The sampling adequacy was good, with a KMO value (Kaiser–Meyer–Olkin) of 0.93. The null hypothesis of Bartlett’s test that the correlation matrix is an identity matrix was rejected (*p* < 0.001). Thus, we were able to perform an EFA on all items. A five-factor structure was found to be the most relevant in light of the scree plot, which showed a plateau in eigenvalues after five factors (see Supplementary Fig. S2). Good fit indices were found for this model (root mean square error of approximation, RMSEA = 0.057 with 90% CI of 0.055–0.058; root mean square of residuals, RMS* r* = 0.04). Results are presented in Table 2 and Fig. 2.

**Table 2:** Loading of items for the five-factor structure.

Items and subscale		Factor					Items and subscale		Factor				
		1	2	3	4	5			1	2	3	4	5
DAS21	EXE	**0.778**	− 0.162	− 0.105	0.062	− 0.059	AMI15	BA	− 0.424	0.115	**0.481**	− 0.012	0.000
MFI11	MF	**− 0.762**	0.294	0.095	0.000	0.080	AMI10	BA	− 0.110	0.154	**0.480**	0.038	0.037
DAS23	EXE	**0.743**	− 0.084	− 0.078	0.118	0.001	MFI6	RA	− 0.268	0.174	**0.415**	− 0.041	0.262
MFI13	MF	**0.733**	− 0.309	− 0.049	0.040	− 0.058	MFI10	RA	0.374	− 0.258	**− 0.406**	0.040	− 0.229
DAS10	EXE	**− 0.703**	0.093	0.280	0.020	0.053	DAS8	INIT	− 0.193	− 0.023	**0.399**	0.144	− 0.044
MFI7	MF	**− 0.687**	0.263	0.196	− 0.012	0.065	MFI15	RM	− 0.089	0.304	**0.380**	0.018	0.286
MFI19	MF	**0.600**	− 0.261	− 0.012	0.118	− 0.146	DAS18	INIT	− 0.258	0.146	**0.290**	0.002	0.240
DAS17	EXE	**0.538**	− 0.106	− 0.269	0.147	− 0.151	AMI18	ES	0.088	0.009	− 0.044	**0.658**	− 0.018
MFI17	MF	**0.511**	− 0.210	− 0.420	0.006	− 0.204	DAS9	EMO	0.074	− 0.064	0.088	**0.658**	0.008
DAS11	EXE	**0.507**	− 0.257	− 0.477	0.032	− 0.197	AMI13	ES	0.021	0.027	− 0.043	**0.601**	0.021
AMI11	BA	**− 0.475**	0.052	0.475	0.029	− 0.064	DAS15	EMO	0.002	0.042	0.023	**− 0.567**	− 0.023
DAS19	EXE	**0.462**	− 0.106	− 0.217	0.088	− 0.142	AMI16	ES	− 0.026	0.060	0.043	**0.548**	0.151
MFI9	RM	**0.385**	− 0.374	− 0.227	0.107	− 0.139	DAS24	EMO	0.052	− 0.040	− 0.033	**− 0.542**	− 0.160
DAS6	EXE	**0.349**	− 0.163	− 0.049	0.042	− 0.192	AMI1	ES	0.065	− 0.180	0.058	**0.531**	0.035
DAS1	EXE	**0.304**	− 0.138	− 0.303	0.243	− 0.166	DAS7	EMO	0.061	− 0.069	0.068	**0.513**	− 0.154
DAS12	EMO	0.285	− 0.042	0.024	− 0.153	− 0.255	DAS5	EMO	− 0.127	0.053	0.118	**0.499**	0.200
MFI1	GF	− 0.196	**0.764**	0.129	− 0.070	0.139	DAS20	EMO	0.058	− 0.076	− 0.058	**0.408**	0.111
MFI5	GF	0.183	**− 0.724**	− 0.011	0.076	− 0.061	AMI7	ES	0.226	− 0.097	0.044	**0.400**	− 0.129
MFI20	PF	− 0.179	**0.714**	0.138	− 0.072	0.107	AMI6	ES	0.328	− 0.159	− 0.039	**0.335**	− 0.244
MFI12	GF	− 0.173	**0.711**	− 0.034	− 0.100	0.063	DAS16	INIT	0.083	− 0.042	0.131	0.166	0.084
MFI14	PF	0.189	**− 0.698**	− 0.122	0.039	− 0.117	AMI14	SM	− 0.215	0.097	0.050	0.014	**0.720**
MFI2	PF	0.217	**− 0.585**	− 0.183	0.013	− 0.128	AMI2	SM	− 0.206	0.097	0.033	− 0.035	**0.676**
MFI3	RA	− 0.227	**0.545**	0.301	− 0.022	0.232	DAS22	INIT	− 0.061	0.012	0.133	0.014	**0.554**
MFI16	GF	0.279	**− 0.519**	− 0.115	0.203	− 0.164	AMI3	SM	− 0.053	0.239	0.010	0.061	**0.479**
MFI8	PF	− 0.187	**0.514**	0.271	− 0.065	0.192	AMI4	SM	− 0.116	0.240	0.184	0.078	**0.454**
MFI18	RM	0.289	**− 0.471**	− 0.297	− 0.036	− 0.250	DAS14	INIT	0.021	0.239	0.378	− 0.046	**0.436**
MFI4	RM	− 0.119	**0.414**	0.258	0.034	0.250	DAS3	EMO	− 0.072	− 0.028	0.033	0.259	**0.404**
DAS13	INIT	− 0.176	0.094	**0.572**	0.072	0.141	DAS2	INIT	− 0.091	0.096	0.156	0.074	**0.403**
AMI12	BA	− 0.450	0.125	**0.516**	− 0.021	0.087	AMI5	BA	− 0.330	0.172	0.271	− 0.282	**0.367**
AMI9	BAB	− 0.461	0.212	**0.494**	− 0.018	0.150	AMI8	SM	0.001	0.165	0.055	0.076	**0.315**
DAS4	INIT	0.066	0.142	**0.488**	0.182	0.265	AMI17	ES	0.006	0.113	0.184	0.041	0.201

The item loadings included into the factor are written in bold.

*AMI* apathy motivation index, *BA* behavioral activation, *ES* emotional sensitivity, *SM* social motivation, *DAS* dimensional apathy scale, *EXE* executive, *INIT* initiation, *EMO* emotional, *MFI* multidimensional fatigue inventory, *GF* general fatigue, *PF* physical fatigue, *MF* mental fatigue, *RA* reduced activity, *RM* reduced motivation.

Factor 1 mostly comprised the items from the DAS executive subscale and the items of the mental fatigue subscale of the MFI, indicating that the executive dimension of apathy and the mental dimension of fatigue share the same latent factor. Factor 2 included only MFI items, mainly those assessing general and physical fatigue. Factor 3 included initiation and behavioral subscales from the DAS and AMI, as well as some items of reduced activity and reduced motivation from the MFI, indicating overlap. Factor 4 comprised only emotional subscales from the AMI and DAS. Factor 5 mostly comprised social items from the AMI and some items of initiation from the DAS.

## Discussion

A lack of energy and a lack of motivation are frequently reported in the healthy population. These conditions can persist over time and have repercussions on daily functioning. Apathy and fatigue may appear to be similar, suggesting an overlap, and this has prompted questions about the characteristics that distinguish them. The aims of this study were to describe the relationship between apathy and fatigue by examining the multidimensions of these two concepts, and to investigate their overlaps and dissociations in a big sample of healthy participants. As expected, converging with previous studies^3,11^, we found several correlations between the subdimensions of apathy and fatigue. The exploratory factor analysis (EFA) revealed overlaps in the reduced activity and mental domains, while the emotional and social domains were found to differ, thus distinguishing apathy from fatigue.

The executive dimension of apathy, representing reduced motivation for organization, attention and planning, showed the strongest correlations with the fatigue scores. The strongest association was found with mental fatigue, representing a feeling of mental lassitude, and both appeared to be underpinned by the same latent factor, according to the EFA. Items from the fatigue questionnaire about reduced activity and reduced motivation were widespread across the factors that support apathy and fatigue (factor 1: executive apathy and mental fatigue, factor 2: general and physical fatigue, factor 3: behavioral apathy). This is consistent with prior knowledge that both apathy and fatigue manifest as reduced activity^1,17–20^. In addition, the overlap of the fatigue items of reduced motivation with the factor that expresses behavioral apathy is also consistent as, conceptually, apathy is defined as a lack of motivation.

Unsurprisingly, the items of initiation and behavioral activation from the AMI and DAS were associated. Further, their latent factor comprised items of reduced activity from the MFI, indicating an overlap. Similarly, the emotional items of the two scales were associated. However, the factor that underpinned emotional apathy did not support any items of fatigue, similar to the last factor of social behavior apathy, indicating that emotional and social components are proper to apathy and do not show specific overlaps with fatigue. This confirms the importance of assessing emotional and social apathy components separately from behavioral components (which mainly manifest through behavioral symptoms, and are more closely associated with fatigue)^1,12^.

Our findings are consistent with results of Ang et al. which reported significant positive correlations between fatigue and behavioral activation as well as social motivation, and negative correlations between fatigue and emotional subdimension of AMI^3^. Our results converge also with those of Jurgelis et al. (2021)^11^. Indeed, we also found correlations between fatigue and executive and action initiation subdimension of DAS. However, in our study we did not found correlations between the emotional subdimension of DAS and MFI scores. Going beyong correlation, in the present study, we employed an EFA to investigate overlaps and differences between apathy and fatigue components. Results showed that apathy and fatigue have reduced activity and mental difficulties in common, whereas emotional and social disorders distinguish apathy from fatigue. In pathological context, similar results were found in studies conducted in Parkinson’s disease, a neurodegenerative disorder with apathy and fatigue as the two of the most common non-motor symptoms. More specifically, significative differences in fatigue were found between apathetic versus non-apathetic patients for the reduced activity, reduced motivation and mental fatigue items^21^, and when apathy was evaluated in fatigued and non-fatigued patients, action initiation and intellectual curiosity but not emotion were found to be significantly different^22^. Furthermore, studies in Parkinson’s disease also showed that apathy and fatigue are separable syndromes^23^. These similarities between results found in healthy participants and different types of patients suggest that similar mechanisms could underlie apathy and motivation in healthy people and pathological populations. This is an interesting area for future investigation.

The similarities between apathy and fatigue may be explained by a shared neurobiological basis. Fatigue is a multifactorial phenomenon and knowledge about its etiology is still limited, but it has been proposed that fatigue may be the consequence of a disturbance in frontal-basal ganglion axis^24,25^. Several studies found that fatigue is associated to high perception of effort and, similarly to apathy, the neurocognitive framework of effort-reward decision making has been proposed to explain at least partially this symptom^12,26^. The anterior cingulate cortex and the ventromedial prefrontal cortex play a role for compute the value of effort and the subsequent effort-based decision making^27^. Studies in fatigue highlighted also the role of sensorimotor and interoceptive information on the effort value which is notably underpinned by the premotor cortex and the insula^7,12^. It has been proposed that these brain regions form together with the dorsolateral prefrontal cortex a circuit in charge of effort-reward decision making, and that the high weighting of effort cost in fatigue may explain the lower engagement in activities. So, disturbances in the prefrontal-basal ganglia loop have been identified for both apathy and fatigue^28,29^. However, evidence suggests that disruption in this brain regions are differently related to the dimensions of apathy. Kumfor et al. (2018) found that alterations in the activity in the left dorsolateral prefrontal cortex are related to the difficulties in elaborating and initiating plans and actions found in cognitive apathy, whereas alterations in the basal ganglia are related to behavioral apathy, and alterations in ventromedial prefrontal cortex are related to emotional blunting^30^. Other neuroimaging studies also found evidence that behavioral, cognitive and emotional dimension of apathy may have distinct neuroanatomical bases^31^.

Behavioral and psychological symptoms such as fatigue, apathy, depression and anhedonia can result in similar observable disorders, making differential diagnosis not trivial. Our findings have important implications for the general population, and may has also implication for neurocognitive and psychiatric disorders. It can help in clinical practice to disentangle symptoms. This have importance as the therapeutic options, in particular pharmacological approaches are different^32^, and treating the wrong symptom may yield deleterious effects^33^. For this reason, it is important to determine the overlaps and distinctions ensure early identification and optimal care.

## Conclusion

This study conducted in the general population showed several correlations between the subdimensions of apathy and fatigue. We found that these disorders have in common reduced activity and mental difficulties, whereas emotional and social disorders allow them to be distinguished. This may have implication for healthy people and may be relevant for subjects with neurocognitive and psychiatric disorders, if a continuity between trait apathy and pathological apathy is demonstrated in future studies. Despite these promising results, several limitations of the study can be noted. First, this study is based uniquely on self-report questionnaires, which carry the risk of subjectivity. Future study should complement self-assessment with behavioral tasks (such as effort-based tasks) and more objective apathy proxies (e.g., the overall level of activity, using actigraphy), to investigate whether new technologies would provide valuable information to complement that provided by the standard clinical instruments, thereby improving differential diagnoses of these symptoms. Second, it would be important to assess whether factors potentially affecting apathy and fatigue (such as depressive symptoms, anxiety, substance use, or the presence of chronic illness) modulate the observed relationship, by adding specific questionnaires assessing those symptoms and pathologies. So, results should be taken with caution because they could not be representative of the general population in its natural state before the pandemic. It would be important to replicate the findings in the future in an independent sample of participants.

## Methods

### Participant/procedure

In all, 729 participants (579 females, 150 males, mean age = 30.8 ± 10.7 years, age range = 18–68) were recruited via social media (Facebook, Twitter, LinkedIn) and a mailing list. The survey was advertised as targeting “healthy adult participants”. We presented information about the study, requested consent to participate, and then asked them to complete anonymous, self-report questionnaires online on the Qualtrics platform. Inclusion criteria was to be over 18 years old. The study was performed in accordance with the Declaration of Helsinki, and approved by the Ethical committee "Comité de Protection des Personnes—CPP Est III, France, MoTap: RCB ID No. 2017-A01366-4". Subjects provided informed written consent for their participation. The data collection was performed between December 2019 and March 2020.

### Questionnaires

Participants were asked to complete three validated questionnaires that assess apathy and fatigue as multidimensional constructs.

Apathy:

- The *Apathy Motivation Index (AMI)*^3^ is a self-administered 18-item scale to assess motivation and apathy in healthy people. Each item is negatively scored from 0 (completely true) to 4 (completely untrue) such that a higher score indicates greater apathy. The AMI is composed of six items for each of three subscales: behavioral activation (goal-directed behavior initiation, e.g., “I get things done when they need to be done, without requiring reminders from others”), social motivation (interest and engagement in social interactions, e.g., “I start conversations without being prompted”) and emotional sensitivity (feeling positive and negative emotions, e.g., “I feel sad or upset when I hear bad news”). We employed the French version of the AMI (f-AMI) validated by Corveleyn and colleagues (submitted). The AMI score were calculated by taking the mean rating of the items within subscales.
- The *Dimensional Apathy Scale (DAS)*^16^ is a self-administered 24-item scale to assess the lack of motivation for planning, organization and attention (executive, e.g., “I am easily distracted”), the lack of emotional motivation (emotional, e.g., “When I receive bad news I feel bad about it”), and the lack of self-generation action (initiation, e.g., “I plan my days activities in advance”). A higher total score indicates more lack of motivation. We employed the French version validated by M’Barek and colleagues (f-DAS, 2020)^34^.

Fatigue

- The *Multidimensional Fatigue Inventory (MFI)*^17^ is a 20-item self-report questionnaire for measuring five dimensions of fatigue: general fatigue (e.g., “I feel tired”), physical fatigue (e.g., “Physically, I feel only able to do a little”), mental fatigue (e.g., “It takes a lot of effort to concentrate on things”), reduced activity (e.g., “I think I do very little in a day”), and reduced motivation (e.g., “I don’t feel like doing anything”). Each subscale contains four items, which are scored on a five-point Likert-scale. Scores range from 4 (absence of fatigue) to 20 (maximum fatigue) for each subscale. We employed the French version validated by Gentile et al. (2003)^35^.

### Statistical analyses

Statistical analyses were performed using R Studio software. Correlational analysis with Pearson’s r and Spearman’s rho were conducted to examine the relationship between all subdimensions of apathy and fatigue. Here, results of Spearman analysis are presented, as data are not normally distributed. We verified whether removing outliers changed the pattern of results. As no difference in the pattern of results was found removing the outliers, we decided to keep all participants in the analyses. Considering the high number of correlations analysis, we applied an alpha level at 0.001. An exploratory factor analysis (EFA) was used to explore the structure of the relationships between variables and allowed to identify latent factors. The EFA was conducted with all items of the three scales after checking results of Kaiser–Meyer–Olkin (KMO) and Bartlett’s tests of sphericity. The Bartlett’s test of sphericity verified whether all correlation coefficients were not null (identity matrix). The KMO tested the sampling adequacy. It informed us about the proportion of common variance and variance that can be explained by other variables. A KMO value better than 0.8 indicate that the sampling is adequate for the EFA. To determine the number of factors, the eigenvalue table and the scree plot were examined. Factors included items with a loading of at least 0.3. Cronbach’s alpha was calculated when an item load was not greater than 0.4 and close to 0.3 to decide whether to include or exclude it.

## Supplementary Information

Supplementary Information.
