# Supplementary material for: Associations, overlaps and dissociations between apathy and fatigue
Source: Sci Rep. 2022 May 5;12:7387. doi: 10.1038/s41598-022-11071-5 (PMC9072543; doi:10.1038/s41598-022-11071-5)
Supplement: Supplementary file 1 — Supplementary Information. [file 41598_2022_11071_MOESM1_ESM.docx]

**SUPPLEMENTARY MATERIAL**

|  | **DAS** | | | | **AMI** | | | | **MFI** | | | | | |
| --- | --- | --- | --- | --- | --- | --- | --- | --- | --- | --- | --- | --- | --- | --- |
|  | **INIT** | **EXE** | **EMO** | **Total** | **BA** | **SM** | **ES** | **Total** | **GF** | **PF** | **MF** | **RA** | **RM** | **Total** |
| Mean | 9.77 | 11.00 | 7.25 | 38.03 | 1.75 | 1.70 | 1.16 | 1.54 | 13.46 | 11.36 | 11.43 | 10.89 | 10.63 | 56.55 |
| Std. Deviation | 3.80 | 5.12 | 3.10 | 4.60 | 0.84 | 0.75 | 0.65 | 0.46 | 4.58 | 4.62 | 4.62 | 4.73 | 3.74 | 18.15 |
| Skewness | 0.17 | 0.25 | 0.74 | -0.80 | 0.17 | 0.21 | 0.75 | 0.27 | -0.38 | 0.13 | 0.10 | 0.33 | 0.25 | 0.06 |
| Minimum | 0.00 | 0.00 | 0.00 | 9.00 | 0.00 | 0.00 | 0.00 | 0.39 | 4.00 | 4.00 | 4.00 | 4.00 | 4.00 | 20.00 |
| Maximum | 22.00 | 24.00 | 22.00 | 50.00 | 4.00 | 3.83 | 4.00 | 3.11 | 20.00 | 20.00 | 20.00 | 20.00 | 20.00 | 100.00 |
|  | | | | | | | | | | | | | | |

**Supplementary Table S1**. Descriptive statistics of data.

AMI=Apathy Motivation Index; BA=behavioral activation; ES=emotional sensitivity; SM=social motivation; DAS=Dimensional Apathy Scale; EXE=executive; INIT=initiation; EMO=emotional; MFI=Multidimensional Fatigue Inventory; GF=general fatigue; PF=physical fatigue; MF=mental fatigue; RA=reduced activity; RM=reduced motivation.

| Apathy questionnaires | **AMI** | | | | **DAS** | | | |
| --- | --- | --- | --- | --- | --- | --- | --- | --- |
|  | **ES** | **BA** | **SM** | **Total** | **EMO** | **INIT** | **EXE** | **Total** |
| **Multidimensional Fatigue Inventory (MFI)** | | | | | | | | |
| **GF** | -.259^*^  (.000) | .393^*^  (.000) | .339^*^  (.000) | .305^*^  (.000) | -.086  (.019) | .263^*^  (.000) | .434^*^  (.000) | .111  (.002) |
| **PF** | -.189^*^  (.000) | .452^**^  (.000) | .381^*^  (.000) | .398^*^  (.000) | .005  (.886) | .334^*^  (.000) | .451^*^  (.000) | .149^*^  (.000) |
| **MF** | -.253^*^  (.000) | .536^*^  (.000) | .314^*^  (.000) | .383^*^  (.000) | .020  (.585) | .300^*^  (.000) | .771^*^  (.000) | .220^*^  (.000) |
| **RA** | -.176^*^  (.000) | .617^*^  (.000) | .414^*^  (.000) | .524^*^  (.000) | .095  (.010) | .483^*^  (.000) | .606^*^  (.000) | .286^*^  (.000) |
| **RM** | -.219^*^  (.000) | .547^*^  (.000) | .438^*^  (.000) | .473^*^  (.000) | .031  (.405) | .452^*^  (.000) | .642^*^  (.000) | .216^*^  (.000) |
| **Total** | -.258^*^  (.000) | .627^*^  (.000) | .471^*^  (.000) | .522^*^  (.000) | .028  (.445) | .466^*^  (.000) | .703^*^  (.000) | .238^*^  (.000) |

**Supplementary Table S2**. Correlations of MFI subscales with AMI and DAS subscales using Pearson’s r.

*Correlation with p<0.001

AMI=Apathy Motivation Index; BA=behavioral activation; ES=emotional sensitivity; SM=social motivation; DAS=Dimensional Apathy Scale; EXE=executive; INIT=initiation; EMO=emotional; MFI=Multidimensional Fatigue Inventory; GF=general fatigue; PF=physical fatigue; MF=mental fatigue; RA=reduced activity; RM=reduced motivation.


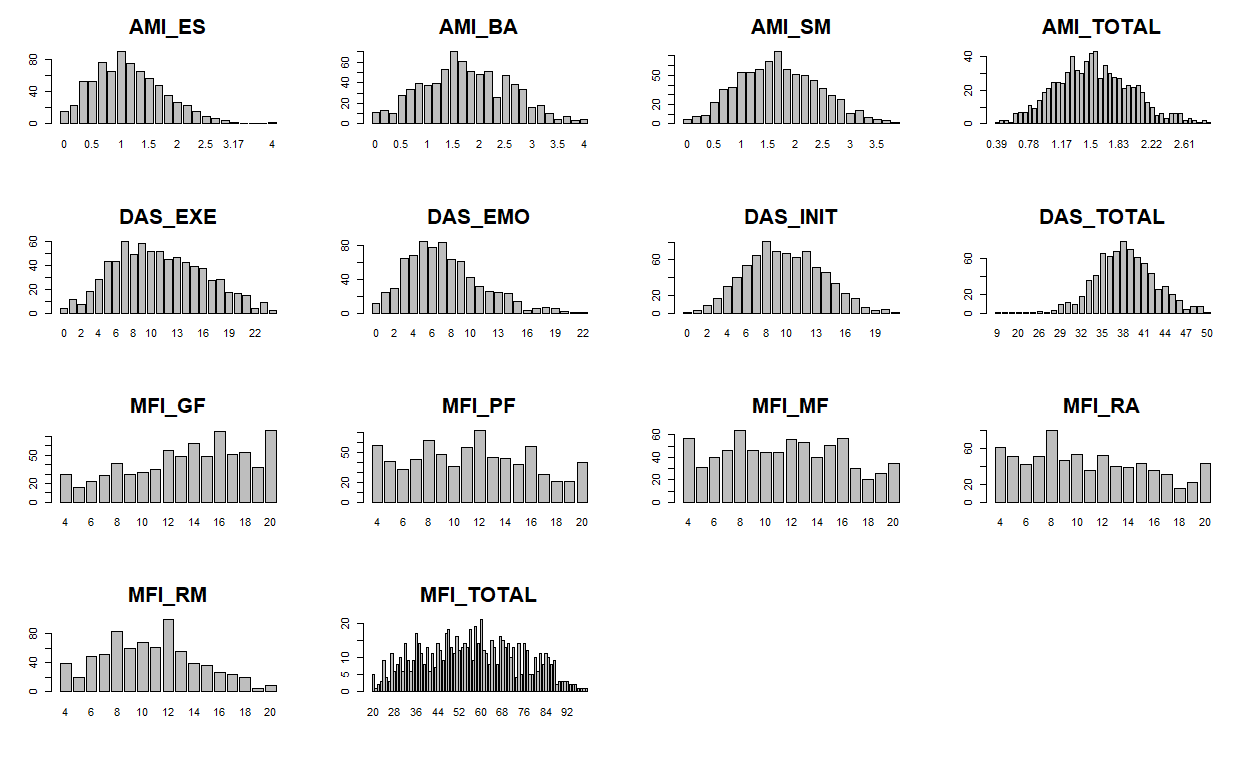


**Supplementary Figure S1**. Frequency histograms.

AMI=Apathy Motivation Index; BA=behavioral activation; ES=emotional sensitivity; SM=social motivation; DAS=Dimensional Apathy Scale; EXE=executive; INIT=initiation; EMO=emotional; MFI=Multidimensional Fatigue Inventory; GF=general fatigue; PF=physical fatigue; MF=mental fatigue; RA=reduced activity; RM=reduced motivation.


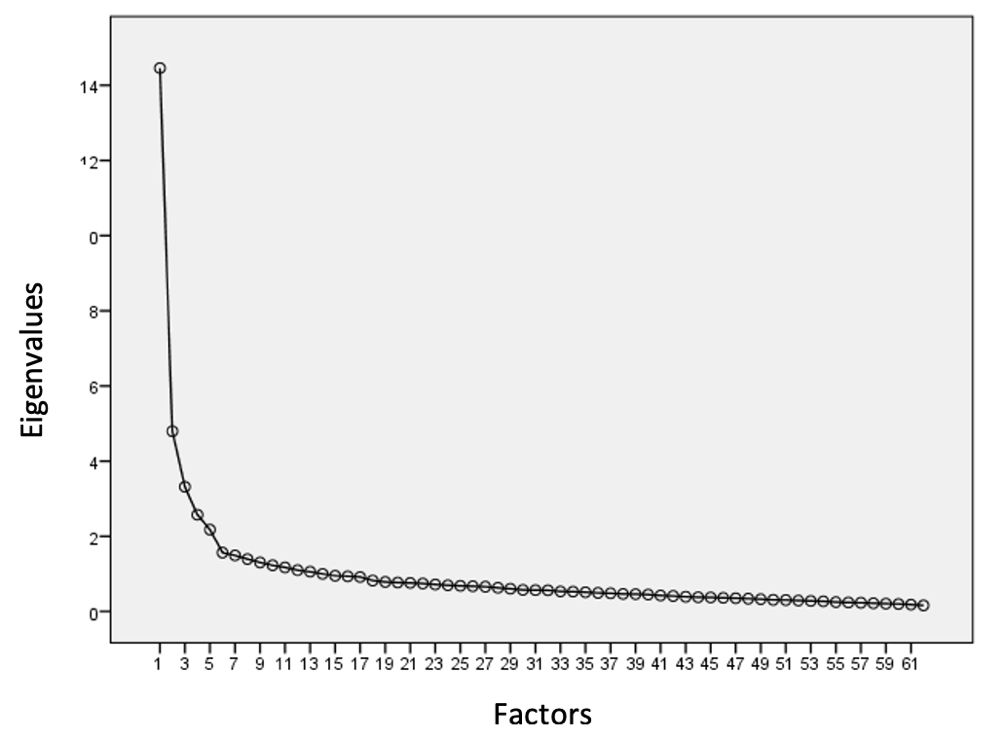


**Supplementary Figure S2.** Scree plot used for deciding the number of factors.
